# From crisis to recovery: how Gaza can overcome its post-war malnutrition challenges

**DOI:** 10.3389/fpubh.2025.1612823

**Published:** 2025-06-10

**Authors:** Abdel Hamid El Bilbeisi, Rana Soboh

**Affiliations:** ^1^Department of Clinical Nutrition, Faculty of Applied Medical Sciences, Al Azhar University of Gaza, Gaza Strip, Palestine; ^2^Department of Nutrition, School of Medicine and Health Sciences, University of Palestine, Gaza Strip, Palestine; ^3^Department of Public Health, Faculty of Public Health, Al-Quds University, Jerusalem, Palestine

**Keywords:** agricultural resilience, food security, Gaza, humanitarian aid, malnutrition, maternal health, nutrition governance, post-war

## 1 Introduction

The Gaza Strip, with its ongoing conflicts, has seen extensive disruptions to its food security and health systems, leading to a rise in malnutrition rates. The ongoing conflict in Gaza has led to a severe humanitarian crisis, severely affecting children's health and nutrition, with over 65,000 children hospitalized due to acute malnutrition caused by food shortages, widespread hunger, and restricted access to essential nutrients. The blockade and destruction of infrastructure have further hindered the delivery of humanitarian aid, worsening food insecurity (1). Additionally, the deliberate denial of food to civilians, particularly vulnerable groups like children, violates international humanitarian law, including the Geneva Conventions, which prohibit using starvation as a method of warfare. It is critical for international organizations to ensure unimpeded access to food and medical supplies, protecting civilians' right to adequate nutrition and humanitarian aid (2). This correspondence discusses the urgent need for a comprehensive approach to combat post-war malnutrition in Gaza, highlighting the role of international aid, local governmental efforts, and sustainable agricultural practices. We present insights from the most recent research on the causes and consequences of malnutrition in Gaza, as well as recommendations for its long-term recovery.

## 2 Discussion

### 2.1 The Gaza Strip's worsening food insecurity and malnutrition crisis

The Gaza Strip, a region under prolonged conflict and blockade, faces severe challenges in ensuring food security and public health. Years of political instability, compounded by recurring wars and economic hardship, have drastically weakened Gaza's food systems. Even prior to the 2023/2025 War, about 71.5% of households in Gaza were already chronic food insecure (3). The latest conflict has significantly worsened conditions. According to the Integrated Food Security Phase Classification (IPC), ~96% of Gaza's population (2.15 million people) are facing acute food insecurity through September 2024 (4). Malnutrition, particularly among children under five, continues to be a major public health issue. Children under five have been disproportionately affected, with skyrocketing rates of malnutrition becoming a pressing public health emergency (5). As these figures show, a multifaceted crisis is unfolding rooted in structural causes and expressed in stark human consequences. This article explores potential solutions to address Gaza's malnutrition challenges post-conflict, offering insights based on current research and potential interventions. Table 1 shows the key nutritional indicators in Gaza (pre- and post-conflict). The results revealed that post-conflict there are an increased levels of chronic food insecurity, acute malnutrition among children under five, access to unsafe water, and displaced population as shown in Table 1.

**Table 1:** Key nutritional indicators in Gaza (pre- and post-conflict).

**Indicator**	**Pre-2023 conflict**	**Post-2023/25 conflict**	**References**
Chronic households food insecurity	71.5%	96%	(3, 4)
Acute malnutrition among children under 5	18%	55%	(6)
Stunting among children under five	25%	Data not yet available	(7)
Population access to unsafe water	~90%	>95%	(8)
Displaced population	~200,000	1.7 million	(9)

### 2.2 Causes of malnutrition in Gaza

Malnutrition in Gaza arises from deeply entrenched structural problems: both man-made and systemic. The principal causes include: (1) Ongoing conflict and economic blockade: since 2007, the blockade has severely limited Gaza's ability to import essential goods such as food, fuel, medical equipment, and agricultural inputs. Repeated military escalations have further damaged critical infrastructure, plunging the economy into crisis and reducing household purchasing power. This has created conditions where food is either unavailable, unaffordable, or inaccessible to large portions of the population (5); (2) Widespread displacement and infrastructure destruction: continuous destruction of housing, hospitals, water systems, and farmland has repeatedly displaced families and undermined public services. With over 1.7 million people displaced in recent escalations (10), access to consistent nutrition and healthcare has been significantly disrupted (11); (3) Collapse of local food systems: the conflict has devastated Gaza's agricultural sector, once a vital lifeline. Damaged greenhouses, loss of livestock, and contaminated soil have led to drastic declines in local food production. Restrictions on fuel and water have further crippled productivity. According to a 2023 cross-sectional study, this systemic breakdown is directly linked to the rise in acute malnutrition among 55% of children surveyed (10); (4) Economic hardship and unemployment: with an unemployment rate exceeding 45%, families are unable to afford sufficient and nutritious food (12). The economic collapse has left many relying solely on humanitarian aid, increasing vulnerability when supply chains are disrupted (13); (5) Poor water quality and sanitation: more than 95% of Gaza's water is unsafe to drink, contributing to waterborne diseases like diarrhea that impair nutrient absorption especially in children (8).

### 2.3 Manifestations and consequences of malnutrition

Malnutrition in Gaza manifests in multiple, interrelated ways that disproportionately affect the most vulnerable, especially children and women. Children under five are among the hardest hit, with acute malnutrition (wasting) causing rapid weight loss and chronic malnutrition (stunting) impairing long-term physical and cognitive development; a study found that over half of Gaza's children suffered from acute malnutrition during the conflict (7). Beyond calorie deficits, widespread micronutrient deficiencies including iron, vitamin A, and iodine further compromise health, particularly among pregnant women and children, increasing susceptibility to disease and heightening risks during childbirth (14). Maternal malnutrition, an often-overlooked crisis, contributes to low birth weight, premature delivery, and long-term developmental challenges in infants. Compounding these issues, the psychological trauma of war marked by displacement, fear, and loss disrupts eating patterns and caregiving routines, making nutritional rehabilitation even more difficult for both children and caregivers under chronic stress (15).

### 2.4 Immediate humanitarian responses

In the immediate aftermath of the 2023/2025 conflict, humanitarian interventions in Gaza must go beyond short-term food relief to restore dignity, address urgent nutritional gaps, and lay the groundwork for sustainable recovery. These responses must be carefully coordinated to function under conditions of ongoing insecurity, logistical limitations, and infrastructural devastation.

#### 2. 4.1 Scaling up nutrition-focused aid with an integrated approach

Emergency food distribution remains vital but this aid must shift from generic calorie-dense packages to nutritionally tailored interventions. Agencies such as the World Food Program (WFP) and United Nations International Children's Emergency Fund (UNICEF) can expand the deployment of ready-to-use therapeutic foods (RUTFs), fortified cereals, and micronutrient powders targeted at children under five, pregnant women, and lactating mothers (16). These interventions should be administered through mobile nutrition units that can reach displaced communities lacking access to clinics. But food alone is not enough. Partnering with local health workers, Non-Governmental Organization (NGOs) can create integrated “nutrition hubs” that bundle food aid with basic health checks, immunizations, and parental education on feeding practices. These community-based hubs should be mobile, allowing flexibility in an unstable security environment. Digital tools like SMS alerts or app-based registration can help track malnutrition cases and ensure consistency in follow-up care despite population displacement.

#### 2.4.2 Jumpstarting agricultural recovery under constraint

While conflict continues to limit access to farmland, recovery must begin even during humanitarian operations. This means providing fast-cycle support to farmers whose assets were destroyed: distributing starter kits with drought-resistant seeds, small-scale solar irrigation pumps, and compact greenhouses that can be deployed even in urban areas or camps. Organizations such as Food and Agriculture Organization (FAO) and International Committee of the Red Cross (ICRC) should prioritize “micro-agriculture” systems that work in constrained environments like rooftop gardens, vertical farming structures, or hydroponic units (17). To support these efforts, international donors must shift from one-time aid shipments toward multi-phase grants that are disbursed as farmers reach specific recovery milestones. In parallel, training programs in sustainable agriculture should be embedded within community spaces to teach families how to grow basic foods and maintain soil health under siege conditions.

#### 2.4.3 Addressing trauma as a nutritional risk factor

Trauma and displacement disrupt more than just shelter, they impact eating behavior, metabolism, and a child's ability to thrive. Humanitarian responses must therefore integrate psychosocial support into nutritional programming. For instance, child-friendly spaces established by NGOs can provide safe environments where trained counselors help children rebuild routines, develop healthy eating behaviors, and receive play-based therapy. At the caregiver level, group-based therapy or stress management workshops should be incorporated into food distribution sites (18). These sessions can be conducted in coordination with local mental health professionals, many of whom continue to operate in Gaza despite resource constraints and should offer coping strategies to help families make the most of limited food resources under intense psychological pressure. Additionally, it is important to recognize mental health as a key factor influencing nutritional outcomes, rather than treating it solely as a standalone humanitarian issue. Incorporating tools like the Patient Health Questionnaire-9 (PHQ-9) or Strengths and Difficulties Questionnaire (SDQ) into nutrition hubs and mobile feeding units can help identify vulnerable families early in the intervention process. Furthermore, integrating psychosocial support with nutrition education such as offering group counseling sessions at food distribution points can yield more effective results than addressing food insecurity alone (18).

### 2.5 Long-term recovery strategies

Recovery in Gaza must be more than a return to pre-war normalcy, it should aim to build food systems that are resilient, locally grounded, and community-led. This requires coordinated investments in people, policies, and infrastructure that address both the nutritional needs and the structural inequalities that fuel food insecurity.

#### 2.5.1 Rebuilding Gaza's agriculture through localized, resilient systems

Reviving Gaza's agricultural capacity requires rethinking production methods to suit the limitations imposed by restricted imports, fuel shortages, and land degradation. Instead of trying to rebuild large-scale agribusiness in the short term, recovery should prioritize localized food systems that can function independently under siege conditions. One promising avenue is the development of urban agriculture corridors designated zones within densely populated areas where community farming can take place. This model has been used successfully in Beirut, Lebanon, where post-conflict urban gardens were implemented by NGOs to promote food security and social cohesion in impoverished neighborhoods (17).

In Syria, humanitarian agencies have introduced micro-agriculture units such as rooftop gardens, vertical farms, and hydroponic systems in cities like Aleppo and Homs, where traditional farmland was inaccessible due to ongoing hostilities (18). These interventions enabled displaced families to grow vegetables in limited spaces with minimal water use, using compact, soil-free techniques supported by solar-powered irrigation systems (19). Lessons from these efforts show that even under aerial threats or shelling, low-cost, modular systems, easily relocated or repaired can help sustain food access.

Gaza can adopt similar strategies by supporting rooftop and courtyard gardens, distributing compact greenhouse kits, and training residents in soilless agriculture and climate-resilient practices. To facilitate these interventions, municipalities and local NGOs could coordinate the development of community-owned seed banks, composting facilities, and shared cold storage spaces. These decentralized systems reduce dependence on long-range transport and offer protection from border closures. To ensure long-term sustainability, international donors should fund youth-focused apprenticeship programs in climate-smart agriculture. These programs, potentially run in partnership with regional agricultural schools or Gaza-based academic institutions, should cover topics such as permaculture, soil regeneration, and organic pest control knowledge that remains viable even in conflict zones.

#### 2.5.2 Community-based nutrition education and food literacy

Improving nutritional outcomes also requires changing what people know and how they eat. Long-term strategies should therefore invest in community-led education campaigns on nutrition, food preparation, and maternal-child health. These campaigns should be developed in collaboration with local NGOs, and educators to ensure cultural alignment. Rather than focusing on formal workshops alone, education can be embedded into everyday spaces markets, mosques, health clinics, and schools. For instance, interactive cooking demonstrations using locally available foods can be held during distribution days. At the school level, integrating nutrition education into core curricula can help cultivate lifelong habits among children who have grown up in crisis (17). Digital tools such as radio programs, WhatsApp groups, and illustrated print materials can broaden reach, particularly among women in conservative or remote areas who may face mobility restrictions.

#### 2.5.3 Empowering women as pillars of nutritional resilience

Women are at the frontline of Gaza's food crisis, often responsible for sourcing, preparing, and rationing food for families. Recovery programs must explicitly recognize this by investing in women's economic and nutritional empowerment. This means providing small business grants and cooperative memberships to women engaged in food production, preservation, or sale. For example, programs can support women-led cooperatives that produce traditional preserved foods, creating both income and local food sources. Reproductive and maternal health services must also be strengthened. Clinics should be equipped not only with maternal supplements but also nutrition counseling, breastfeeding support, and anemia screenings especially during the early postpartum period, where the risk of undernutrition is highest (20). These women-centered interventions should be part of a broader effort to mainstream gender equity in recovery planning, ensuring that women are involved in decision-making, monitoring, and implementation of food systems reform.

### 2.6 Policy and advocacy recommendations

Addressing Gaza's food insecurity and malnutrition crisis also requires policy changes and robust advocacy. However, the feasibility of many proposed interventions depends heavily on the political, security, and infrastructural context on the ground. For instance, (1) Lifting the blockade and easing restrictions remains a cornerstone of long-term recovery, but achieving this goal depends on sustained international pressure and political negotiations that may face resistance. Even partial easing must be accompanied by mechanisms that ensure transparency and prevent the re-imposition of restrictions during escalations (21). (2) International diplomatic efforts must not only secure the flow of humanitarian aid but also create conditions for rebuilding civilian infrastructure without triggering military retaliation. Without durable ceasefires or diplomatic guarantees, many projects like rebuilding farms or establishing rooftop gardens face high risk of destruction. (3) Local government and international collaboration must be supported by enhanced accountability mechanisms to ensure aid reaches communities most in need and is not undermined by factional divides or administrative fragmentation. Realistic planning should include contingency strategies for recurring conflict and emergency shutdowns of food supply routes.

Several barriers could impede the success of solutions to Gaza's malnutrition crisis, including political instability, ongoing conflict, the blockade, coordination issues, limited funding, and logistical challenges like damaged infrastructure and restricted access. These obstacles hinder the effective delivery of humanitarian aid. While international assistance is essential, it can foster dependency and be influenced by political factors that delay aid. To overcome these challenges, efforts should focus on strengthening Gaza's agricultural infrastructure, improving local governance, and empowering communities through education and skill-building, ensuring that aid complements initiatives to enhance local production and sustainability, promoting long-term self-reliance and recovery.

Additionally, NGOs and international aid actors are vital in addressing Gaza's malnutrition crisis, but they face challenges from donor fatigue and politicized aid. Without lasting political solutions, donor interest may decline, and funding decisions can become tied to geopolitical agendas rather than need. This risks delays, conditional aid, and loss of trust. To maintain effectiveness, NGOs must ensure transparent, needs-based aid and strengthen local capacity to reduce reliance on fluctuating external support.

### 2.7 Strengthening governance for nutrition security in Gaza

Strengthening governance is vital for achieving lasting nutrition security in Gaza, as humanitarian aid alone cannot resolve underlying structural challenges. The authors emphasize the need for decentralized decision-making, stronger institutional capacity, and inclusive governance. This includes empowering local bodies, forming community nutrition committees, and fostering leadership among women and youth. They advocate for international support to enhance policy development, nutrition monitoring, and supply chains. A “resilience governance” approach focused on long-term planning, cross-sector collaboration, and contingency strategies is essential. Resilience governance is the coordination of structures and processes that help societies anticipate, adapt to, and transform in response to shocks, while maintaining core functions. It merges resilience thinking with governance theory, focusing on flexibility, learning, inclusivity, and multi-level collaboration (22). Inclusive mechanisms like participatory budgeting and youth councils are also crucial to ensure community voices shape nutrition and health outcomes.

### 2.8 Stakeholder roles for effective nutrition recovery in Gaza

The roles of local government, international organizations, and civil society in Gaza's post-war malnutrition recovery is essential for coordinated, accountable, and sustainable intervention. Local governments are best positioned to facilitate access, policy integration, and infrastructure support, acting as key enablers of both emergency response and long-term systems reform. International organizations bring critical funding, technical expertise, and global coordination capacity, which are especially vital in times of acute crisis and when rebuilding health, food, and agricultural systems. Civil society including NGOs, community groups, and local health workers serves as the critical bridge between strategy and impact, executing programs on the ground, mobilizing community participation, and ensuring culturally relevant service delivery. By delineating these roles more explicitly within each strategy from nutrition aid to agricultural recovery and mental health support, stakeholders can reduce duplication, fill gaps more effectively, and improve the efficiency of both humanitarian and developmental efforts. In a fragile and dynamic context like Gaza, such role clarity is not merely strategic, it is foundational to saving lives and building resilience.

## 3 Conclusion

The post-war malnutrition crisis in Gaza represents a complex humanitarian emergency rooted in structural inequalities, repeated conflict, and governance challenges. While short-term humanitarian aid remains essential, achieving lasting recovery necessitates a shift toward inclusive, resilient, and community-driven solutions. However, it is critical to acknowledge the considerable barriers that may limit the feasibility of some interventions. For instance, localized agriculture solutions such as rooftop farming or vertical gardens offer promise in theory, but their viability in a war-prone environment especially one vulnerable to aerial strikes remains uncertain. Similarly, the ability to maintain mobile nutrition hubs or deploy agricultural technology assumes a level of safety, mobility, and infrastructure that may not always be present. Thus, recovery efforts must balance ambition with realism, incorporating contingency plans and risk assessments. True progress will require not only technical innovation and strong governance but also the political will to address the root causes of Gaza's vulnerability. With inclusive decision-making, gender-sensitive planning, and flexible, conflict-aware programming, Gaza can move toward a more secure and dignified future though the path will be neither easy nor linear.
